# Maternal cafeteria diet and methyl donor supplementation modulate gut microbiota and anxiety-like behavior in male offspring

**DOI:** 10.29219/fnr.v70.14244

**Published:** 2026-06-29

**Authors:** Ximena Rodríguez Gómez, Heriberto Castro, Alberto Camacho-Morales

**Affiliations:** 1 Universidad Autonoma de Nuevo León, Centro de Investigación en Nutrición y Salud Pública, Facultad de Salud Pública y Nutrición, Monterrey, México; 2 Universidad Autonoma de Nuevo León, Departamento de Bioquímica, Facultad de Medicina, Monterrey, México

**Keywords:** hypercaloric diet, social behavior, microbial diversity, intestinal environment, intrauterine development, anxiety-related disturbances

## Abstract

Maternal cafeteria diet and methyl donor supplementation modulate gut microbiota and behavioral outcomes in male offspring.

**Background:**

Maternal hypercaloric diets rich in saturated fats and refined sugars are associated with metabolic alterations, gut microbiota dysbiosis, and behavioral disturbances in offspring. Methyl donor supplementation has been proposed as a potential strategy to modulate these effects.

**Objective:**

To evaluate the effect of maternal cafeteria diet consumption and methyl donor supplementation on gut microbiota composition and behavioral outcomes in male offspring in a murine model.

**Design:**

Female C57BL/6 mice were assigned to four dietary groups: control, cafeteria diet, control supplemented with methyl donors, and cafeteria diet supplemented with methyl donors. Diet exposure occurred during pre-gestation, gestation, and lactation. Male offspring were evaluated at 8 weeks of age using behavioral tests and gut microbiota analysis based on 16S rRNA gene sequencing. Associations between gut microbiota and behavioral parameters were evaluated using adjusted linear regression models controlling for maternal diet and methyl donor supplementation.

**Results:**

Offspring from cafeteria-fed dams supplemented with methyl donors showed higher microbial diversity compared to the non-supplemented cafeteria group. Cafeteria diet increased the abundance of *Deferribacteres, Mucispirillum, Adlercreutzia, Butyricicoccus*, and *Prevotella*, whereas methyl donor supplementation reduced Deferribacteres and modified the abundance of *Paraprevotella* and *Ruminococcus_1*. At the species level, *Mucispirillum schaedleri* and *Lactobacillus reuteri* were increased under cafeteria dietary conditions. No significant effects were observed in sociability-related variables after adjustment. However, *Butyricicoccus* remained associated with central and peripheral zone behavior, whereas *Paraprevotella* remained positively associated with wall-leaning behavior after adjustment for maternal diet and supplementation status.

**Discussion:**

Maternal cafeteria diet modulated offspring gut microbiota composition and was associated with anxiety-related behavioral parameters. Methyl donor supplementation showed differential effects depending on maternal dietary context, reducing specific bacterial taxa associated with inflammatory and metabolic alterations.

**Conclusions:**

Maternal methyl donor supplementation attenuated specific microbiota alterations induced by cafeteria diets and was associated with microbiota–behavior relationships related to anxiety-like responses in offspring.

## Popular scientific summary

- Earlier studies indicate that maternal diet influences offspring behavior, including sociability and anxiety.
- No changes were observed in sociability, but specific microbiota were associated with anxiety-like behavior.
- Methyl donor supplementation altered gut microbial composition associated with behavioral changes.
- Maternal diet influenced offspring outcomes differently depending on dietary conditions.

A hypercaloric diet is characterized by its high content of saturated fats and refined sugars, an example of these being Western or cafeteria-type diets (1). The components of these diets adversely impact both maternal health and the offspring through a phenomenon known as maternal programming. Among the adverse metabolic alterations resulting from exposure to hypercaloric diets, specifically in the offspring, are effects on fetal adiposity, inflammatory state, insulin resistance, and disruption of energy homeostasis, among others (2). On the other hand, there is a close link between the quality of the maternal diet and the modulation of the fetal gut microbiota, so hypercaloric diets contribute to establishing gut dysbiosis that may be harmful not only for intrauterine development, but also in postnatal stages (3). Studies reveal that the increase in *Firmicutes* promotes weight gain by altering energy absorption mechanisms (4). At the same time, the decrease in the population of *Lactobacillus spp*. causes greater permeability of the intestinal barrier, which leads to a systemic inflammatory state (5); both effects are induced by diets high in saturated fats. In addition to the impact at the microbiota level, the effects may extend even to behavior, as high-fat diets have been associated with social behavior disorders and anxiety-related disturbances (6). As possible modulators of developmental programming, methyl donor-supplemented diets have been proposed. There is evidence indicating that early intake of methyl donors is associated with alterations in cognition and behavior, and that these effects may involve gut microbiota-related pathways and one-carbon metabolism processes previously associated with gene expression and neurodevelopment (7, 8). Previous work from our group, using the same maternal dietary model, showed that cafeteria diet exposure modified behavioral parameters in male offspring, while methyl donor supplementation partially modulated these responses. However, the microbial mechanisms potentially underlying these results remain unclear, and the biological effects of methyl donor supplementation may depend on the dose, timing of exposure, and maternal metabolic context, as both deficiency and excessive intake have been associated with adverse outcomes (9–11). Therefore, the aim of the present study is to evaluate the effect of maternal consumption of a cafeteria diet and supplementation with methyl donors on offspring gut microbiota and behavioral outcomes in a murine model. We hypothesized that maternal methyl donor supplementation would attenuate cafeteria diet-induced alterations in gut microbiota composition and modulate microbiota–behavior associations in offspring.

## Materials and methods

### Experimental model and diets

The present study was derived from a previously established maternal programming model reported by our research group (9), in which behavioral alterations induced by maternal cafeteria diet and methyl donor supplementation were originally characterized in male offspring. In the current study, the same experimental cohort was further analyzed to evaluate gut microbiota composition and microbiota–behavior associations.

Table 1 shows the experimental diets used during the maternal intervention. Dams received one of the following dietary treatments: (1) standard chow diet (CTL), containing 3.35 kcal/g (Rodent Lab Chow diet 5001; LabDiet, St. Louis, MO, USA); (2) cafeteria diet (CAF), containing 3.72 kcal/g and composed of liquid chocolate, fried potatoes, bacon, biscuits, standard chow diet, and pork paté in a 1:1:1:1:1:2 ratio, respectively; (3) standard chow diet supplemented with methyl donors (CTL + SUP); and (4) cafeteria diet supplemented with methyl donors (CAF + SUP). Supplemented diets contained betaine (5 g/kg diet), choline (5.37 g/kg diet), folic acid (5.5 mg/kg diet), and vitamin B12 (0.5 mg/kg diet) (12–15).

**Table 1:** Composition of the experimental diets and methyl donor supplementation

Component	CTL diet (kcal/kg diet)	CAF diet (kcal/kg diet)	Methyl donor supplementation(per kg diet)
Total energy	3,350	3,720	-
Carbohydrates	1,909	1,450	-
Protein	1,005	446.6	-
Lipids	435.5	1,822	-
Choline	-	-	5.37 g
Betaine	-	-	5 g
Vitamin B9 (Folic acid)	-	-	5.5 mg
Vitamin B12	-	-	0.5 mg

Values for carbohydrates, protein, and lipids correspond to the caloric contribution (kcal/kg diet) of each macronutrient. CTL: control diet; CAF: cafeteria diet.

### Animals and experimental design

Virgin female C57BL/6 mice (11 weeks old) (Scientific, Technological, and Commercial Services S.A. de C.V., Monterrey, Mexico) were housed in polypropylene cages under controlled environmental conditions (21–22°C; 12-h light/dark cycle) with ad libitum access to food and water. Following a 1-week acclimatization period, females were randomly assigned to four maternal dietary groups: control diet (CTL, *n* = 3 dams), cafeteria diet (CAF, *n* = 5 dams), control diet supplemented with methyl donors (CTL + SUP, *n* = 5 dams), and cafeteria diet supplemented with methyl donors (CAF + SUP, *n* = 3 dams).

**Fig. 1:**
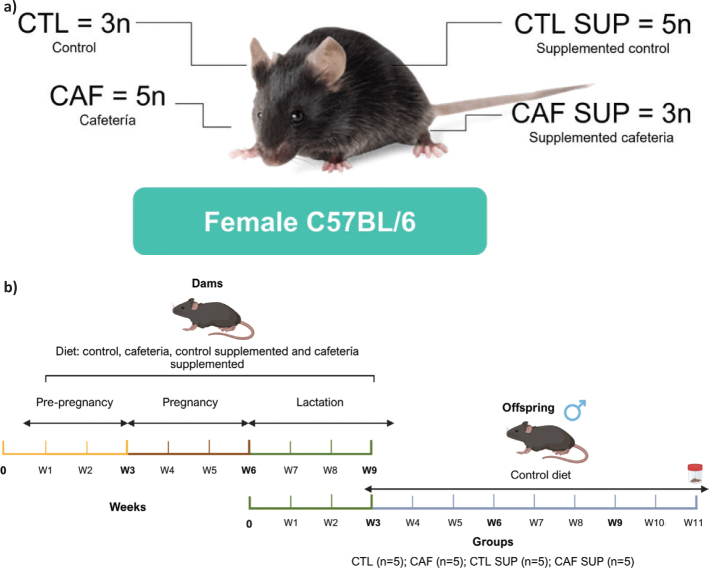
Experimental design and dietary intervention. Female C57BL/6 mice were assigned to four maternal dietary groups: CTL (*n* = 3 dams), CAF (*n* = 5 dams), CTL SUP (*n* = 5 dams), and CAF SUP (*n* = 3 dams), during pre-pregnancy, pregnancy, and lactation. Male offspring were subsequently evaluated under a control diet. CTL: control diet; CAF: cafeteria diet; SUP: supplemented.

Maternal dietary interventions were maintained for 9 weeks, including pre-gestation, gestation, and lactation periods. Females were mated with age- and strain-matched males using one male per female. Pregnancy was confirmed by the presence of a vaginal plug. After weaning at postnatal day 21, male offspring were maintained under standard chow conditions until 8 weeks of age (Fig. 1).

Behavioral phenotyping was previously performed in male offspring using the three-chamber test (3CT) and open-field test (OF), as reported in the original experimental study (9). For the current analyses, gut microbiota sequencing was performed using a subset of male offspring (*n* = 5/group) from the previously characterized cohort, and previously generated behavioral data were used for microbiota–behavior association analyses. Only animals with both behavioral and microbiota data available were included in these analyses.

Behavioral phenotyping was previously performed in male offspring using the 3CT and OF, as reported in the original experimental study (9), and these previously generated behavioral data were used for microbiota–behavior association analyses in the present study. In the present work, gut microbiota sequencing analyses were performed using a subset of male offspring (*n* = 5/group) from the previously characterized cohort. Only animals with both behavioral and microbiota data available were included in microbiota-behavior association analyses.

### Behavioral phenotyping

Behavioral procedures were conducted as previously described by Herrera et al. (8). Briefly, social interaction behavior was evaluated using the 3CT (16), whereas locomotion, exploratory activity, and anxiety-like behavior were assessed using the OF (17). Behavioral parameters analyzed in the present study included sociability-related variables, time spent in central and peripheral zones, and wall-leaning behavior.

### Microbiota analyses

#### Fecal collection

After sacrifice at week 11, feces were collected from each group (Control, Cafeteria, Supplemented Control, and Supplemented Cafeteria, *n* = 5/group). The samples were placed in sterile 15-mL Falcon tubes and kept frozen at -80°C until further analysis (18).

#### Deoxyribonucleic Acid (DNA) extraction and quantification

For the bacterial DNA extraction, the QIAamp Fast DNA Stool Mini Kit (Qiagen, Germany) was used according to the manufacturer’s instructions. Subsequently, the samples were stored at -20°C. A NanoDrop8000 spectrophotometer (Thermo Scientific) was used to determine the quality of the bacterial DNA.

#### Amplification of the 16S Ribosomal RNA (rRNA) gene

The first step consisted of the amplification of the V3 and V4 regions of the 16S rRNA gene using the Polymerase Chain Reaction (PCR) with specific primers for these regions, which also contain an adapter sequence for the Illumina Nextera XT indices. Thus, the complete primer sequences used were:

Forward: 5’TCGTCGGCAGCGTCAGATGTGTATAAGAGACAGCCTACGGGNGGCWGCAG 3’. Reverse:5’ GTCTCGTGGGCTCGGAGATGTGTATAAGAGACAGGACTACHVGGGTATCTAATCC 3’

### Statistical analysis

Evaluation of the diversity and composition of the gut microbiota of offspring:

The Kruskal–Wallis test with Bonferroni as post hoc was used for nonparametric data, or its parametric equivalent, one-way Analysis of variance (ANOVA) with Tukey’s test as post hoc. The data were analyzed using Statistical Package for the Social Sciences (SPSS) statistical software version 25.0, considering *P* < 0.05 as significant. In addition, the identification and graphical representation of the set of bacterial phyla, genera, and species was performed using the Interactive Tree of Life (iTOL, https://itol.embl.de/) platform.

Analysis of associations between offspring gut microbiota and behavioral parameters: The Kruskal–Wallis test with Bonferroni as post hoc was used for nonparametric data, or its parametric equivalent, one-way ANOVA with Tukey’s test as post hoc. Associations between gut microbiota abundance and behavioral parameters were subsequently evaluated using adjusted linear regression models that included maternal diet and methyl donor supplementation as covariates. Standardized regression coefficients (Std. *β*) and *P* values were calculated using SPSS statistical software version 25.0, considering *P* < 0.05 as statistically significant.

## Results

### Impact of maternal diet on offspring intestinal bacterial composition

**Fig. 2:**
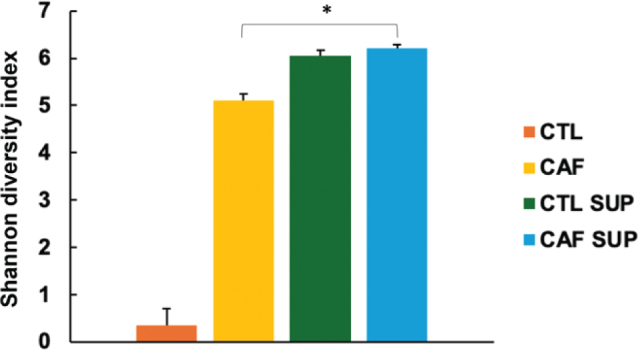
Alpha diversity is represented by the Shannon index. Results are expressed as mean ± standard deviation. Statistical analysis was performed using a nonparametric Kruskal–Wallis test followed by Dunn’s post hoc test. *P < 0.01. Control groups (CTL; *n* = 5 offspring/group), cafeteria (CAF; *n* = 5 offspring/group), supplemented control (CTL SUP; *n* = 5 offspring/group), and supplemented cafeteria (CAF SUP; *n* = 5 offspring/group). CTL: control diet; CAF: cafeteria diet.

The Shannon index revealed differences in bacterial diversity among maternal dietary groups. Offspring from the cafeteria diet supplemented with methyl donors group showed higher diversity values compared to the non-supplemented cafeteria group (*P* < 0.002) (Fig. 2).

**Fig. 3:**
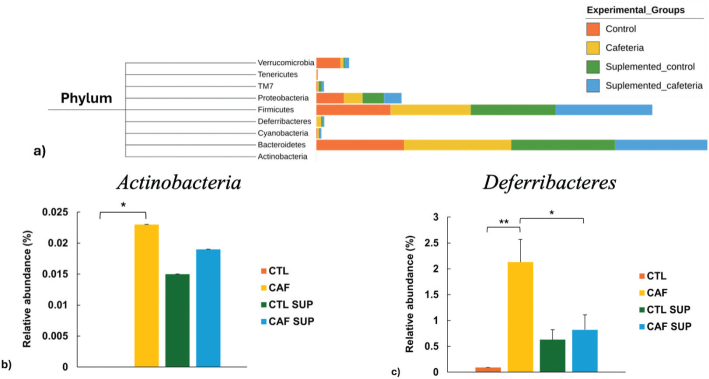
Relative bacterial abundance (%) of the intestinal microbiota at the phylum level in male C57BL/6 mice. (a) Identified phyla set, (b) Actinobacteria, and (c) Deferribacteres. Only phyla showing statistically significant differences between maternal dietary groups are displayed individually in panels b and c due to their potential biological relevance. Results are expressed as mean ± standard deviation (*n* = 5 offspring/group). Statistical analysis was performed according to data distribution: the Kruskal–Wallis test followed by Dunn’s post hoc test was used for nonparametric data, and a one-way ANOVA with Tukey’s Honestly Significant Difference (HSD) test was used for parametric data. *P < 0.05, **P < 0.01. Control, cafeteria, supplemented control, and supplemented cafeteria groups.

At the taxonomic level of phylum, a significant difference was found in bacteria such as *Actinobacteria* and *Deferribacteres*, with higher relative abundance levels in the cafeteria groups compared to the control groups (*P* < 0.040 and *P* < 0.001, respectively) (Fig. 3). Furthermore, a reduction in *Deferribacteres* was observed in the offspring of cafeteria dams supplemented with methyl donors compared to the non-supplemented cafeteria group.

**Fig. 4:**
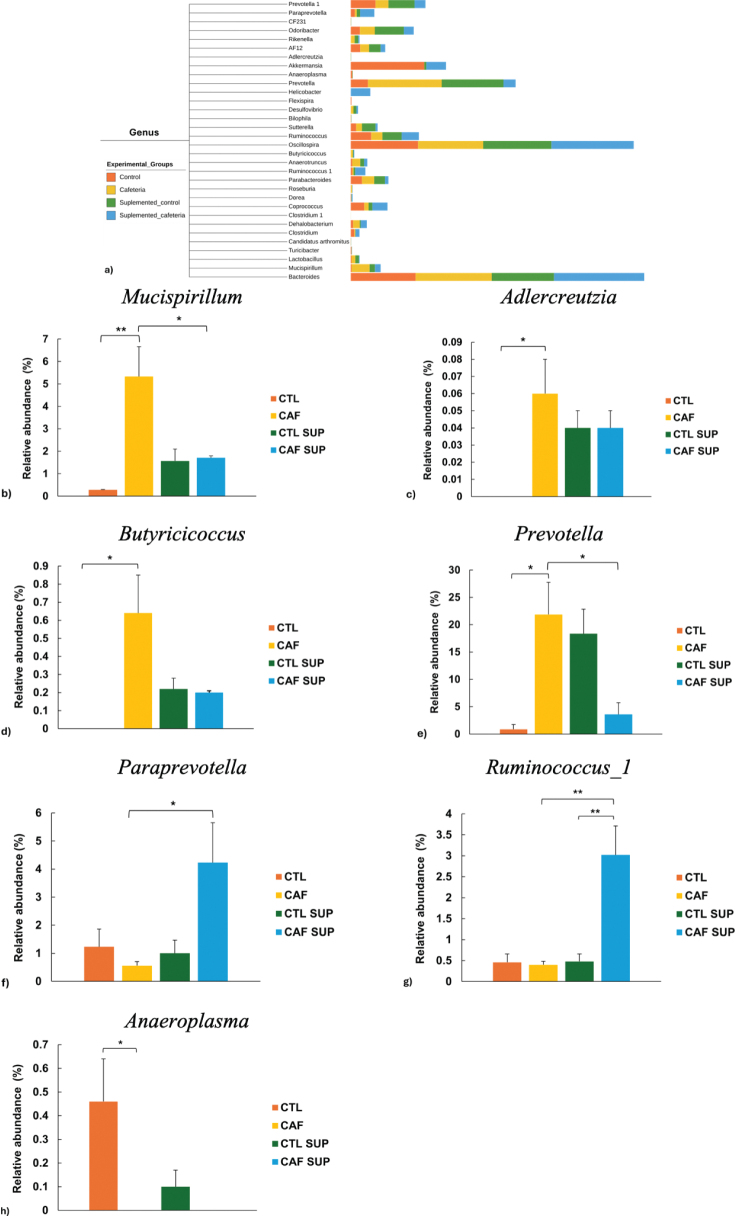
Relative bacterial abundance (%) of the intestinal microbiota at the genus level in male C57BL/6 mice. (a) Identified genera set, (b) *Mucispirillum*, (c) *Adlercreutzia*, (d) *Butyricicoccus*, (e) *Prevotella*, (f) *Paraprevotella*, (g) *Ruminococcus_1*, and (h) *Anaeroplasma*. Results are expressed as mean ± standard deviation (*n* = 5 offspring/group). Statistical analysis was performed according to data distribution: the Kruskal–Wallis test followed by Dunn’s post hoc test was used for nonparametric data, and a one-way ANOVA with Tukey’s HSD test was used for parametric data. *P < 0.05, **P < 0.01. Control, cafeteria, supplemented control, and supplemented cafeteria groups.

Regarding the taxonomic classification at the genus level, the offspring of dams fed a cafeteria diet showed an increase in bacteria such as *Mucispirillum* (*P* < 0.001), *Adlercreutzia* (*P* < 0.04), *Butyricicoccus* (*P* < 0.01), and *Prevotella* (*P* < 0.04) compared to the control group. Furthermore, an opposite effect was observed in *Anaeroplasma*, with a higher presence in the offspring of dams fed the control diet than in those fed the cafeteria diet (*P* < 0.02) (Fig. 4). Moreover, *Mucispirillum* differed not only from the control diet, but also from the supplemented cafeteria diet (*P* < 0.01). Additionally, offspring whose dams consumed cafeteria diets supplemented with methyl donors presented elevated levels of *Paraprevotella* and *Ruminococcus_1* (*P* < 0.05, *P* < 0.001, respectively), compared to the non-supplemented cafeteria diets.

Bacteria such as *Prevotella* and *Mucispirillum* also predominated in high-fat diets, such as the cafeteria diet, compared to the cafeteria diet supplemented with methyl donors (*P* < 0.01 and *P* < 0.02, respectively). In contrast, *Ruminococcus_1* showed higher levels under a supplemented cafeteria diet compared to a supplemented control diet (*P* < 0.001).

**Fig. 5:**
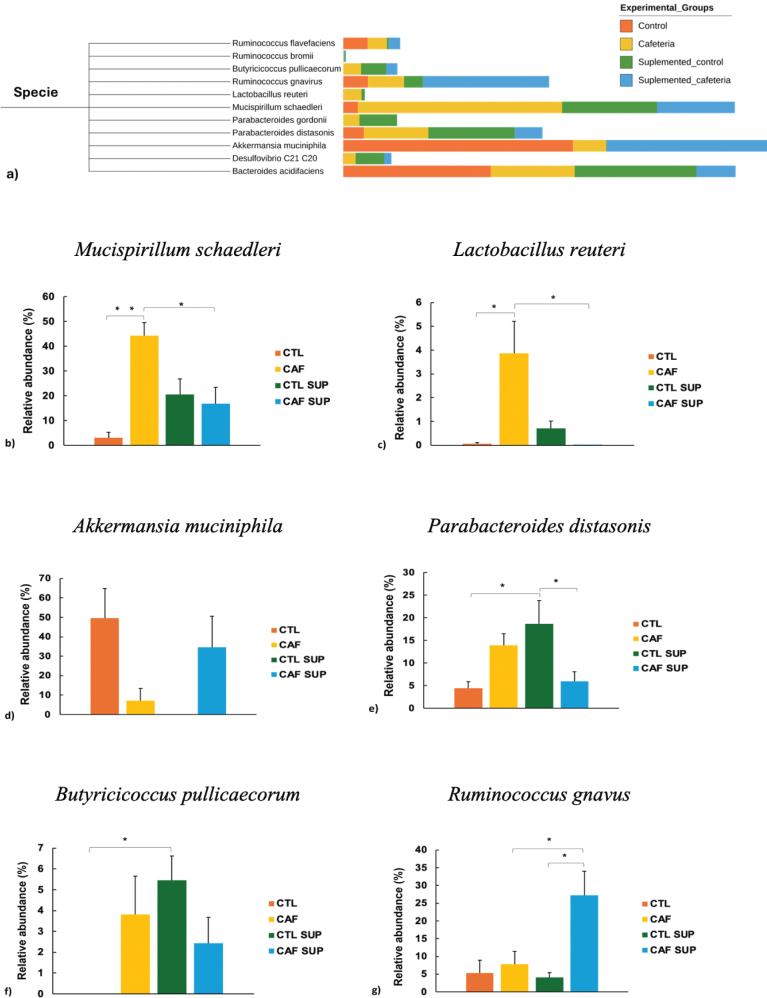
Relative bacterial abundance (%) of the intestinal microbiota at the species level in male C57BL/6 mice. (a) Identified species set, (b), (c), (d), (e), (f), (g). Results are expressed as mean ± standard deviation (*n* = 5 offspring/group). Statistical analysis was performed according to data distribution: the Kruskal–Wallis test followed by Dunn’s post hoc test was used for nonparametric data, and a one-way ANOVA with Tukey’s HSD test was used for parametric data. *P < 0.05, **P < 0.01. Control, cafeteria, supplemented control, and supplemented cafeteria groups.

At the species level (Fig. 5), offspring from dams in the cafeteria group showed a higher abundance of *Mucispirillum schaedleri* compared to the supplemented cafeteria group (*P* < 0.01) and the control group (*P* < 0.001). For its part, *Lactobacillus reuteri* showed a higher abundance under a cafeteria-type diet compared to a control diet and a supplemented cafeteria diet (*P* < 0.006 and *P* < 0.007). Conversely, *Akkermansia muciniphila* showed a trend of higher abundance in the control group compared to the cafeteria group and the supplemented control group; however, it did not reach statistical significance (*P* > 0.054). A higher abundance was observed in the supplemented control groups relative to the control groups for bacteria such as *Parabacteroides distasonis* and *Butyricicoccus pullicaecorum* (*P* < 0.02 and *P* < 0.04, respectively). Similarly, for *P. distasonis*, it indicated a greater presence under a supplemented control diet compared to a supplemented cafeteria diet (*P* < 0.02). Furthermore, the abundance of *Ruminococcus gnavus* increased in the supplemented cafeteria group compared to the supplemented control group (*P* < 0.008) and the cafeteria group (*P* < 0.03). These findings indicate that maternal methyl donor supplementation was also associated with selective microbial modulation under standard dietary conditions, although these effects appeared less pronounced than those observed under cafeteria dietary exposure.

### Offspring gut microbiota and its relationship with behavior

Previous work by our research group has demonstrated that maternal cafeteria-type diets and methyl donor supplementation modulate social interaction and anxiety-like behavior in male offspring using behavioral paradigms such as three-chamber and OF tests. Building on these previously identified behavioral alterations, the present study aimed to evaluate potential associations between the offspring gut microbiota and selected behavioral parameters. To reduce potential confounding effects from maternal dietary interventions, adjusted linear regression models were performed that included maternal diet and methyl donor supplementation as covariates.

No significant associations were observed for variables related to sociability behavior after adjustment for maternal diet and methyl donor supplementation. Therefore, subsequent analyses focused on OF behavioral parameters associated with anxiety-like behavior. Likewise, no significant associations were detected between *Butyricicoccus pullicaecorum* abundance and sociability-related variables, including attraction to a novel object and sniffing behavior.

**Fig. 6:**
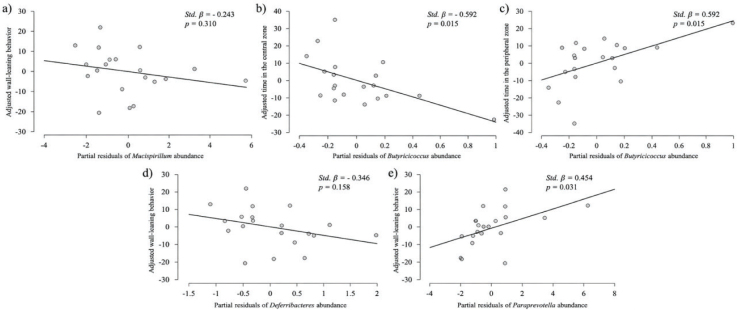
Partial regression plots of gut microbiota taxa associated with behavioral outcomes in male offspring after adjustment for maternal diet and methyl donor supplementation. (a) *Mucispirillum* vs. wall-leaning behavior; (b) *Butyricicoccus* vs. time spent in the central zone; (c) *Butyricicoccus* vs. time spent in the peripheral zone; (d) *Deferribacteres* vs. wall-leaning behavior; and (e) *Paraprevotella* vs. wall-leaning behavior. Solid lines indicate adjusted linear regression models. Standardized *β* coefficients and *P* values are shown in each panel (*n* = 5 offspring per maternal dietary group; total *n* = 20).

Within OF behavioral parameters, adjusted linear regression analyses identified significant associations for *Butyricicoccus* abundance. Specifically, a negative association was observed with time spent in the central zone (Std. *β* = −0.592, *P* = 0.015) (Fig. 6b), whereas a positive association was detected with time spent in the peripheral zone (Std. *β* = 0.592, *P* = 0.015) (Fig. 6c). Furthermore, negative associations were observed between wall-leaning behavior and the genera *Deferribacteres* (Std. *β* = −0.346, *P* = 0.158) (Fig. 6d) and *Mucispirillum* (Std. *β* = −0.243, *P* = 0.310) (Fig. 6a); however, these associations were not statistically significant after adjustment for maternal diet and methyl donor supplementation. At the species level, *Mucispirillum schaedleri* showed a similar tendency.

In contrast, *Paraprevotella* abundance showed a positive association with wall-leaning behavior (Std. *β* = 0.454, *P* = 0.031) (Fig. 6e). Additional non-significant associations were observed for other OF behavioral parameters under different maternal dietary conditions (see Supplementary Table S1). Adjusted linear regression models evaluating microbiota).

The adjusted regression models identified specific bacterial taxa associated with OF behavioral parameters after controlling for maternal diet and methyl donor supplementation. In particular, *Butyricicoccus* and *Paraprevotella* remained significantly associated with anxiety-related behavioral measures, whereas associations observed for *Mucispirillum* and *Deferribacteres* were attenuated after adjustment. These findings support a potential association between specific gut microbial taxa and behavioral alterations in offspring under different maternal dietary conditions, including methyl donor supplementation.

## Discussion

Diet is a factor that can directly influence gut bacterial diversity, as measured by the Shannon index. We found a higher Shannon diversity index in offspring from the cafeteria diet supplemented with methyl donors group compared to the non-supplemented cafeteria group. In this context, greater diversity has been positively associated with improvements in metabolic health (19), potentially through mechanisms involving short-chain fatty acid production, intestinal homeostasis, and inflammatory regulation (20). Based on our results, supplementation with methyl donors appears to have a positive effect in the presence of a hypercaloric diet, such as the cafeteria diet, but not under standard dietary conditions. A 2018 study in mice reported that deficiencies in folate and choline negatively impact offspring health by altering intestinal development and reducing bacterial diversity (21). However, it remains unclear whether these effects result from a direct action of methyl donors on bacteria or from secondary changes in the intestinal environment that favor microbial diversity. Under standard dietary conditions, offspring may present a relatively stable microbial environment, potentially limiting the modulatory effects of methyl donor supplementation. Therefore, the effects of supplementation may become more evident under metabolically adverse conditions characterized by microbial dysbiosis and inflammatory alterations (22).

The obesogenic diet produces gradual effects on the microbial environment and consequently on gut microbiota composition. At the phylum level, *Deferribacteres* and *Actinobacteria* showed higher relative abundance in the cafeteria group than in the control group, whereas *Adlercreutzia* and *Mucispirillum* were increased at the genus level. Previous studies have associated *Deferribacteres* with obesity induced by high-fat diets, intestinal inflammation, insulin resistance, and elevated BMI (23–25). Similarly, higher levels of *Mucispirillum* schaedleri have been reported under high-fat dietary conditions and proposed as a marker of intestinal inflammation (24, 26). Together, these findings support the association between hypercaloric diets and microbial taxa previously linked to inflammatory and metabolic alterations.

Furthermore, both *Deferribacteres* and *Mucispirillum* showed reduced relative abundance following methyl donor supplementation, suggesting that supplementation may attenuate microbial taxa previously associated with hypercaloric diet-induced inflammatory and metabolic alterations (21).

In contrast, *Ruminococcus* and *Ruminococcus gnavus* showed increased relative abundance under maternal hypercaloric dietary conditions despite methyl donor supplementation, particularly in the cafeteria supplemented group. *R. gnavus* has previously been associated with dysbiosis, inflammatory conditions, and dyslipidemias (26, 27). These findings suggest that some bacterial taxa associated with adverse metabolic profiles may persist under obesogenic dietary conditions despite supplementation (28). Han et al. reported a significant association between *R. gnavus* and elevated folate levels in humans (29). Likewise, experimental studies in mice have proposed that *R. gnavus* may metabolize choline into trimethylamine (TMA), a precursor of pro-inflammatory metabolites (30, 31) which could partially explain the increased abundance of these taxa in supplemented cafeteria groups. Collectively, these findings suggest that metabolites derived from folate and choline may contribute to microbial conditions favoring the persistence of these bacteria under hypercaloric dietary exposure.

*Anaeroplasma* showed higher abundance in the control group. Although research on this genus remains limited, it has been associated with alterations in intestinal morphology, high-fat diet intake, obesity, and inflammation (32–34). In contrast, *Prevotella* and *Butyricicoccus*, which have been associated with standard or reduced-fat dietary patterns (35, 36), showed greater abundance under cafeteria dietary conditions, suggesting a possible microbial response to adverse metabolic environments. *Prevotella* also appeared to respond positively to methyl donor supplementation, supporting a potential modulatory effect of these compounds on gut microbiota composition under hypercaloric conditions.

*Lactobacillus reuteri* exhibited a distinct abundance pattern, with higher levels observed in the cafeteria group and lower levels following methyl donor supplementation. Previous studies have associated *L. reuteri* with favorable metabolic outcomes in obesity and diabetes mellitus (37, 38). Therefore, the increased abundance observed under cafeteria dietary conditions may reflect a compensatory microbial response to inflammatory alterations induced by high-fat, high-sugar diets. Similar increases in *L. reuteri* under metabolically adverse conditions have been previously reported, whereas its abundance tends to remain more stable under standard dietary conditions (39). Collectively, these findings support the influence of maternal dietary environment on microbial taxa previously associated with favorable metabolic functions.

Previous studies have reported that *Paraprevotella* decreases under hypercaloric dietary conditions and may be restored by Mediterranean diets or prebiotic compounds (40, 41). In the present study, *Paraprevotella* abundance increased in offspring from cafeteria-fed dams supplemented with methyl donors, whereas lower abundance was observed in the non-supplemented cafeteria group, consistent with previous associations between reduced *Paraprevotella* levels and high-fat intake (42). Likewise, Parabacteroides distasonis and *Butyricicoccus pullicaecorum* have been associated with beneficial metabolic functions, including succinate and butyrate production, which may contribute to intestinal and metabolic homeostasis (43, 44).

Previous studies have associated *Akkermansia muciniphila* with favorable metabolic profiles and reduced obesity risk (45). However, no significant differences in *A. muciniphila* abundance were observed among groups in the present study.

Gut microbiota composition has been associated with anxiety-related behavioral responses through gut-brain axis signaling pathways (46). In the present adjusted analysis, *Butyricicoccus* abundance remained associated with OF variables related to anxiety-like behavior after adjustment for maternal diet and methyl donor supplementation. Previous work from our research group demonstrated increased anxiety-like behavior in offspring from cafeteria-fed dams (9). Although *Butyricicoccus* has been associated with butyrate production and favorable metabolic functions, its role in behavioral regulation remains uncertain. Short-chain fatty acids such as butyrate have been implicated in intestinal signaling, neurotransmitter modulation, and serotonergic pathways (43, 47) suggesting a potential relationship between *Butyricicoccus* abundance and anxiety-related behavioral alterations.

*Deferribacteres* and *Mucispirillum* have previously been associated with inflammatory and metabolic alterations (48). In the present study, maternal methyl donor supplementation reduced the relative abundance of these taxa under cafeteria dietary conditions. However, the associations initially observed between these bacteria and wall-leaning behavior were attenuated after adjustment for maternal diet and supplementation status, suggesting that maternal dietary environment may act as an important confounding factor underlying these behavioral associations.

Previous work from our research group demonstrated that methyl donor supplementation modulated anxiety-like behavioral parameters in offspring, including OF exploratory behavior such as wall leaning (9). Maternal cafeteria diets have also been associated with anxiety-related and neurodevelopmental alterations in offspring (49, 50), whereas previous work derived from the same experimental model identified alterations in circulating galanin levels, a neuropeptide associated with anxiety-related and neuropsychiatric responses (51, 52). Although epigenetic and serotonergic pathways have been proposed in maternal nutritional programming models (43, 44), the present study did not directly evaluate these mechanisms.

In contrast, *Paraprevotella* abundance increased under methyl donor-supplemented cafeteria diets and remained positively associated with wall-leaning behavior after adjustment for maternal diet and supplementation status, suggesting that some bacterial taxa may retain independent associations with anxiety-related behavioral parameters despite maternal dietary interventions. Choline metabolism has previously been associated with microbial TMA production and subsequent trimethylamine-N-oxide (TMAO) formation (53), whereas microbiota-derived metabolites have been linked to neurobehavioral responses, including anxiety-related parameters (54). In addition, *Paraprevotella* has been associated with folate metabolism and microbial pathways involved in host physiological regulation (55).

Based on the present findings, maternal cafeteria-type diets induced alterations in offspring gut microbiota composition and were associated with anxiety-related behavioral alterations previously identified in this experimental model. Adjusted regression analyses identified specific bacterial taxa, particularly *Butyricicoccus* and *Paraprevotella*, that remained associated with OF behavioral parameters after controlling for maternal diet and methyl donor supplementation. These findings support a potential relationship between maternal dietary environment, gut microbiota composition, and anxiety-related behavioral responses in offspring.

One limitation of this study is that only male offspring were evaluated to reduce variability associated with sex-dependent behavioral and hormonal differences previously reported in maternal programming and microbiota–behavior interaction studies. Therefore, female offspring may exhibit distinct microbial and behavioral responses to maternal diet and methyl donor supplementation. In addition, offspring from maternal dietary groups were analyzed as biological replicates, which may not fully account for potential litter-specific effects.

## Conclusions

Supplementation with methyl donors is presented as a potential strategy to mitigate the adverse effects of maternal hypercaloric diets. Our results highlight the possible beneficial effects of this supplementation in an adverse environment, such as hypercaloric diets, including attenuation of microbial dysbiosis and modification of metabolic-related indicators in the offspring. However, it would be important to determine whether methyl donors act directly on the bacteria or whether their effects are mediated indirectly, creating a more favorable intestinal environment. Nevertheless, certain bacterial taxa previously associated with adverse metabolic profiles, such as *Ruminococcus gnavus*, exhibited increased abundance despite methyl donor supplementation under cafeteria dietary conditions. Furthermore, this study suggests that the presence of a single bacterium alone is not a decisive factor for generating adverse metabolic or neuronal effects. Rather, the negative effects could be a combination of factors, including microbial imbalance, translating into an altered metabolic status. On the other hand, these findings may reflect maternal diet-related programming effects on offspring gut microbiota composition. Alterations in microbial taxa associated with maternal dietary conditions, including *Anaeroplasma*, may influence the relative abundance of other taxa previously associated with favorable metabolic functions, such as *Lactobacillus reuteri* and *Prevotella*. Therefore, *Anaeroplasma* may represent a microbial taxon of interest associated with maternal high-fat and high-sugar dietary conditions. Additionally, maternal methyl donor supplementation was associated with behavioral responses related to anxiety-like traits in offspring, while associations observed for taxa such as *Deferribacteres* and *Mucispirillum* were attenuated after adjustment for maternal diet and supplementation status. Finally, although several bacterial taxa identified in the present study have previously been associated with favorable metabolic functions, alterations in their relative abundance may also be associated with behavioral changes under specific maternal dietary conditions. Metabolites derived from gut microbiota, including short-chain fatty acids and folate-related compounds, have been proposed as potential modulators of gut-brain communication; however, the mechanisms underlying these associations were not directly evaluated in the present study.

## Supplementary Material


