# Supplementary material for: Maternal cafeteria diet and methyl donor supplementation modulate gut microbiota and anxiety-like behavior in male offspring
Source: Food Nutr Res. 2026 Jun 29;70:10.29219/fnr.v70.14244. doi: 10.29219/fnr.v70.14244 (PMC13325957; doi:10.29219/fnr.v70.14244)
Supplement: Supplementary file 1 [file FNR-70-14244-s1.docx]

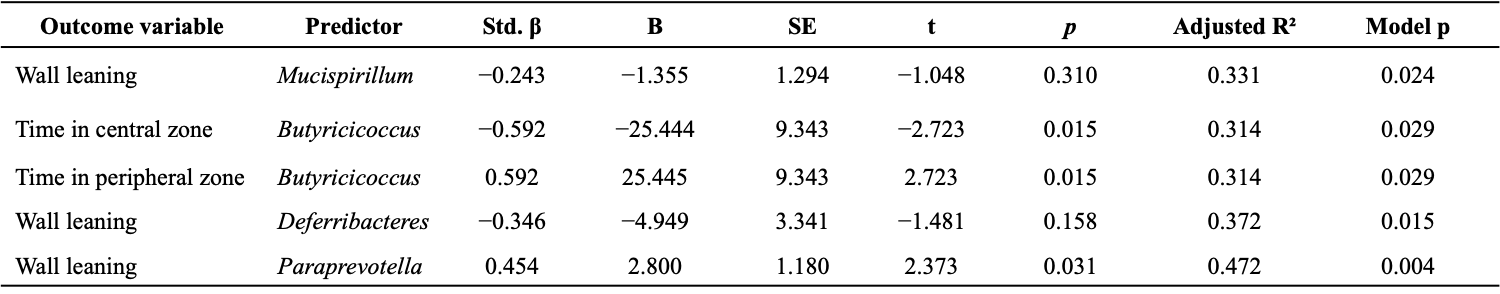


Multiple linear regression models were adjusted for maternal diet and methyl donor supplementation. Std. β represents standardized regression coefficients. Significant associations are indicated in bold.

Supplementary Table S1. Adjusted linear regression models evaluating microbiota - behavior associations in offspring
